# Report from the 9th Italian Society for Virology (SIV-ISV) 2025 Annual Meeting

**DOI:** 10.3390/v18060684

**Published:** 2026-06-18

**Authors:** Anna De Filippis, Manuela Donalisio, Anna Luganini, Francesca Caccuri, Francesca Esposito, Nicole Grandi, Carla Zannella, Luisa Rubino, Enzo Tramontano, Gabriele Vaccari, Massimiliano Galdiero, Arnaldo Caruso

**Affiliations:** 1 Department of Woman, Child and General and Specialized Surgery, University of Campania Luigi Vanvitelli, 80138 Naples, Italy; anna.defilippis@unicampania.it (A.D.F.); carla.zannella@unicampania.it (C.Z.); massimiliano.galdiero@unicampania.it (M.G.); 2 UOC Virology and Microbiology, University Hospital “Luigi Vanvitelli”, 80138 Naples, Italy; 3 Department of Clinical and Biological Sciences, University of Turin, 10043 Orbassano, Italy; manuela.donalisio@unito.it; 4 Department of Life Sciences and Systems Biology, University of Turin, 10123 Turin, Italy; anna.luganini@unito.it; 5 Department of Molecular and Translational Medicine, University of Brescia, 25123 Brescia, Italy; francesca.caccuri@unibs.it; 6 Department of Life and Environmental Sciences, Cittadella Universitaria di Monserrato, University of Cagliari, 09045 Cagliari, Italy; francescaesposito@unica.it (F.E.); nicole.grandi@unica.it (N.G.); tramon@unica.it (E.T.); 7 Consiglio Nazionale delle Ricerche, Istituto per la Protezione Sostenibile delle Piante, Sede Secondaria di Bari, 70126 Bari, Italy; luisa.rubino@cnr.it; 8 Department of Food Safety, Nutrition and Veterinary Public Health, Istituto Superiore di Sanità, 00161 Rome, Italy; gabriele.vaccari@iss.it

**Keywords:** SIV-ISV, virology, One-Health, virus–host interaction, environmental virology, food virology, antiviral therapy, plant virology, conference report

## Abstract

The 9th National Congress of the Italian Society for Virology (SIV-ISV), entitled “One Virology—One Health”, took place in Turin at the Centro Congressi Lingotto from 22 to 24 June 2025. The meeting highlighted recent multidisciplinary and translational developments in virology, with a strong focus on the integration of the One Health perspective. Major themes included viral emergence and surveillance, genomic sequencing and bioinformatics, virus–host interactions, viral immunology and vaccines, structural and physical virology, environmental and food virology, zoonoses and animal infections, diagnostics and antiviral therapy, virus-based biotechnology and plant virology. The Congress aimed to: (i) bring together clinicians, basic researchers, veterinarians, environmental microbiologists, bioinformaticians, public-health professionals and industry to share methodologies and best practices; (ii) provide an interactive scientific environment promoting discussion and collaboration between senior investigators and trainees through plenaries, joint society sessions, invited talks, oral communications selected from abstracts, poster sessions, and mentoring panels; and (iii) identify priorities and inspire new research directions at the interface of human, animal and environmental health. More than 400 participants from national and international institutions attended the meeting, featuring distinguished plenary speakers, joint sessions with global networks, and numerous presentations of original unpublished data. This report summarizes the meeting’s scientific highlights, cross-disciplinary discussions, and proposed actions to strengthen One Health surveillance, computational infrastructures, and translational applications of viral biology.

## 1. Introduction

The 9th National Congress of the Italian Society for Virology (SIV-ISV), “One Virology—One Health”, convened in Turin from 22 to 24 June 2025 at the Centro Congressi Lingotto (Via Nizza 280). The meeting assembled a wide and diverse community—academic and clinical virologists, veterinarians, environmental and ecological microbiologists, bioinformaticians, public health professionals, representatives from regulatory bodies and industry, and a large contingent of early career researchers and students—united by the shared recognition that viruses shape health, society, and science in multiple, interconnected ways. The Congress took place against the background of recent global infectious emergencies, which have made vividly clear that viral threats rapidly transcend species, ecosystems, and national borders, and that effective preparedness and response require integrated One Health frameworks combining human, animal, and environmental perspectives. At the same time, several talks showed that viruses themselves are indispensable research tools and platforms: from viral vectors applied in gene therapy and vaccinology to virus-based systems for studying molecular assembly, immunity, and oncogenic processes, the discipline of virology now operates at the intersection of basic biology, translational medicine, and biotechnology.

The Congress has been organized under the presidency of Arnaldo Caruso and chaired by Rossana Cavallo, Cristina Costa, Giorgio Gribaudo, and David Lembo, with a scientific secretariat formed by Marco De Andrea, Manuela Donalisio, and Anna Luganini, which together with the SIV-ISV Executive Board (Enzo Tramontano, Massimiliano Galdiero, Luisa Rubino, and Gabriele Vaccari) oversaw program and policy. The Congress offered a comprehensive program designed to foster cross-disciplinary dialogue, highlight recent advances, and catalyze new collaborative initiatives. The planned program combined plenary sessions, joint society symposia, notably the SIV-ISV/AMCLI joint session, invited keynote lectures, thematic sessions, and parallel tracks. These covered a broad spectrum of virology: viral immunology and vaccines; virus–host interactions and cell death pathways; structural virology and biotechnology; environmental and food virology; viruses and cancer; zoonoses and animal infections; diagnostics in human virology; antiviral therapy; emerging and reemerging viruses; and plant virology. Each session integrated basic, translational, and applied perspectives to reflect the modern practice of virology, which spans molecular mechanisms and population-level surveillance, laboratory diagnosis, and public health implementation.

The Congress placed special emphasis on genomic surveillance and virus bioinformatics, topics that have become central as sequencing and high-throughput metagenomic methods produce ever larger datasets. Dedicated talks and roundtables addressed how to adapt analytical tools to the specific needs of virology, the development and governance of cloud-based pipelines, assessment of computational resources, and mechanisms for rapid and secure cross-border data sharing. Participants discussed concrete priorities: the standardization of metadata, harmonization of pipelines for clinical and environmental sequencing, integration of genomic data with epidemiological and ecological information, and training programs to build bioinformatics skills among virologists. These discussions recognized that scalable computational infrastructures and well-designed data sharing policies are prerequisites for timely detection of variants, real-time surveillance of pathogen spread, and coordinated One Health responses.

Plenary lectures and joint sessions exemplified the Congress’s translational and One Health focus. International and national speakers examined the effects of climate change and ecosystem alteration on pathogen emergence and transmission dynamics; presented advances in sequencing technologies and genomic epidemiology for respiratory viruses including SARS-CoV-2, Influenza, and RSV; summarized lessons from viral vectored vaccine development and their implications for future vaccine platforms; and reported structural biology studies elucidating mechanisms of viral entry, assembly, and antigenicity. Sessions on environmental monitoring and food safety highlighted methods for wastewater and foodborne virus surveillance, risk assessment frameworks, and the integration of environmental virology into public health decision-making. Equally, sessions on viruses and cancer bridged clinical oncology and viral pathogenesis, showcasing biomarker development, serology-based screening strategies, and translational research on HPV related cancers. Animal health and zoonosis sessions—covering topics such as hepatitis E, lentiviral evolution in small ruminants, and the animal reservoirs of emerging pathogens-reinforced the message that surveillance and control strategies must span species barriers and ecological contexts.

The program balanced high-profile invited talks with presentations selected from submitted abstracts, enabling a large portion of the scientific community to contribute. Oral sessions drawn from submitted abstracts offered visibility to new results, while extensive poster sessions provided occasions for in-depth, face-to-face discussion. Scholarships, travel grants, and awards, funded in part by sponsors and society resources, were allocated to maximize trainee participation and to encourage geographical and institutional diversity among presenters.

The social and networking components of the meeting played an important role in seeding collaborations and fostering community. Extended coffee breaks, poster viewing sessions, a faculty dinner, and a social dinner created informal settings for exchange; these convivial moments often spawned project ideas, inter-institutional partnerships, and mentoring relationships. The Organizing Committee intentionally designed the schedule to maximize opportunities for such interactions while preserving time for in-depth scientific discussion. The SIV-ISV Award 2025 and other recognitions celebrated outstanding contributions across career stages and disciplines, emphasizing both scientific excellence and leadership within the virology community.

The attendance reflected wide participation from Italy and abroad, with delegates representing universities, research institutes, clinical centers, public health agencies, and industry partners. This mixture of stakeholders enriched debates and ensured that sessions addressed both fundamental research questions and operational challenges.

Importantly, about 101 young virologists participated in the event and sent 82 abstracts. Finally, we warmly thank the Editors of “Viruses” for supporting the publication of a Special Issue also entitled “Virology in Italy 2025—9th National Congress of the Italian Society for Virology,” and that will bring together this meeting report and peer-reviewed original articles and reviews. We are confident that this Special Issue will be attractive to all, including those of our colleagues who could not attend the 9th SIV-ISV 2025 National Congress, and that it will represent a comprehensive source of information for all interested in virology.

## 2. Conference Sections: Selected Invited Lectures

### 2.1. Session 1: Viral Immunology and Vaccines—Chaired by Roberto Burioni, Roberta Rizzo, and Carolina Scagnolari (Figure 1)

On the inaugural day, in Session 1 entitled “Viral Immunology and Vaccines”, Prof. Raul Andino (Department of and Immunology, University of California, USA) held a lecture on “Live-attenuated vaccines: virus evolution, immunity, and protection”, in which he first reminded the key progress and facts in the eradication of poliovirus. Since 1988, global efforts have led to a 99.9% reduction in wild poliovirus cases. At that time, polio was endemic in over 125 countries with around 350,000 cases annually. Today, wild poliovirus remains endemic in only two countries: Afghanistan and Pakistan. Of the three poliovirus serotypes, types 2 and 3 were declared eradicated in 1999 and 2020, respectively. Only wild poliovirus type 1 is still circulating. Vaccination efforts have prevented paralysis in over 20 million people and saved more than 1.5 million lives. These achievements are largely due to the Global Polio Eradication Initiative (GPEI), a partnership led by national governments and six key organizations: WHO, Rotary International, CDC, UNICEF, the Bill & Melinda Gates Foundation, and Gavi. GPEI strategies and tactics encompass high immunization coverage, robust surveillance, rapid response to outbreaks, and community engagement. However, several challenges hinder global polio eradication efforts: access and security, mobile and hard-to-reach populations, vaccine hesitancy and misinformation, weak health systems, and cross-border coordination. Prof. Andino pointed out the advantages of the Oral Polio Vaccine (OPV) versus the Inactivated Polio Vaccine (IPV). OPV offers several key benefits over IPV, including community protection (it not only protects individuals but also stops virus transmission), ease of use, early protection of newborns through maternal antibodies, herd immunity, and broader immunity due to stimulation of innate immunity that may protect against other viruses. He described the molecular bases of de-attenuation of OPV, pointing out that only 3 mutations drive the initial path to virulence (2 in the 5’UTR and 1 in capsid protein VP1). Studies have revealed that mutations accumulate fast post-vaccination, as soon as 14 days after vaccination with trivalent OPV, supporting a model in which an increase in fitness follows a defined evolutionary pathway [1]. RNA viruses, those replicating via an RNA-dependent RNA polymerase, exhibit high mutation rates and frequent recombination. Insertions and deletions have a pivotal role in virus evolution, emergence, and regulation of viral infection [2]. Genetic recombination is frequent among human enteroviruses, including polioviruses, EV-A71, EV-D68, and coxsackieviruses. These RNA viruses recombine both intra- and inter-specifically, facilitating the emergence of novel variants. Notably, recombination between attenuated OPV strains and circulating non-polio enteroviruses, such as asymptomatic coxsackieviruses, can generate circulating Vaccine-Derived Polioviruses (cVDPVs), representing a key obstacle to polio eradication [3]. Indeed, both mutations and recombination play a central role in the emergence of cVDPV, by replacement of viral sequences where attenuation determinants map, with similar sequences generated de novo by mutations or by recombining with co-circulating enteroviruses that do not possess attenuating determinants. The emergence of cVDPVs, particularly from Sabin type 2 strains, poses a major challenge to global PV eradication. To counteract these threats, the team directed by Prof. Andino has developed novel oral poliovirus vaccine type 2 (nOPV2) candidates incorporating polymerase mutations and changes to an RNA structure that reduce evolutionary potential. In detail, an RNA structure within the 5’UTR region involved in attenuation of virus replication in neurons (domain V) was stabilized, the cis-acting replication element (cre) was relocated to the 5’UTR, and two amino acid substitutions in the RNA polymerase were introduced to improve fidelity. Clinical trials have demonstrated that nOPV2 is safe and immunogenic. Since March 2021, nOPV2 has been deployed in outbreak responses under the World Health Organization’s Emergency Use Listing (EUL). Additionally, the WHO reported that between March 2021 and December 2023, approximately 1.3 billion doses of nOPV2 were administered across around 41 countries, in nearly 250 outbreak response campaigns. In December 2023, nOPV2 received full licensure from Indonesia’s regulatory agency (Badan POM) and achieved WHO Prequalification, marking a significant step toward broader use [4,5]. Future strategies for PV vaccine amelioration encompass rationally engineering live attenuated virus vaccines. The tissue-specific expression patterns of microRNAs (miRNAs), and the RNA interference (RNAi) response can be exploited to control virus replication in tissues where the virus can cause disease. Poliovirus strains engineered to become the target of neuro-specific miRNAs have been shown to replicate in the central nervous system and exhibit significantly attenuated neurovirulence, even though retaining the ability to replicate in non-neuronal tissue. Notably, production of high levels of neutralizing antibodies has been reported, confirming effective immune responses [6]. These innovative strategies could contribute to the Polio Eradication program.

**Figure 1:**
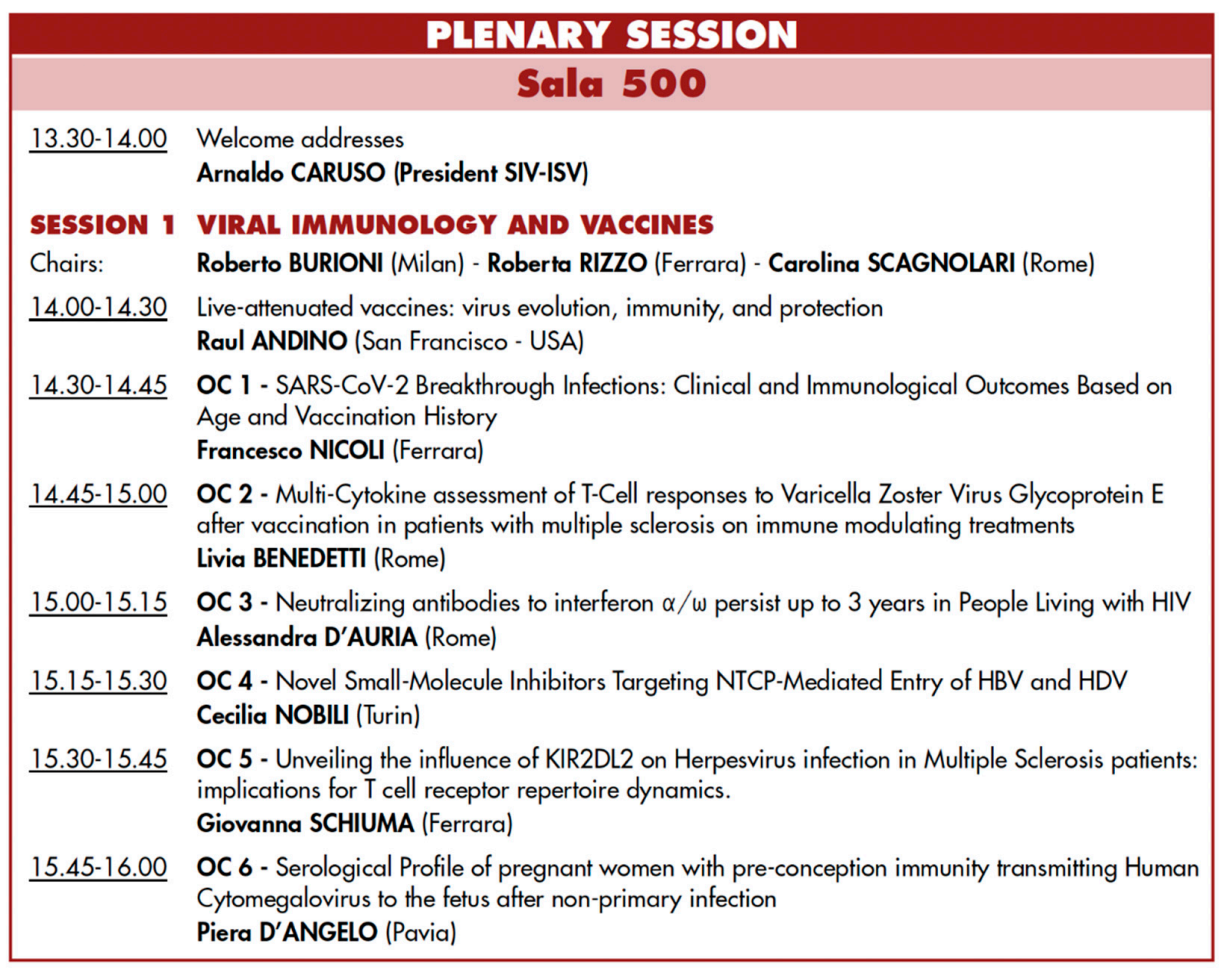
Session 1.

### 2.2. Session 2: Viral Pathogenesis and Diseases—Chaired by Fausto Baldanti and Giovanni Giammanco (Figure 2)

With the lecture “Facing the Rising Dengue Challenges,” Prof. Luisa Barzon (Department of Molecular Medicine, University of Padua, Italy) kicked off Session 2, “Viral pathogenesis and diseases” of the SIV ISV 2025, demonstrating the critical issues that have emerged in recent years related to the Dengue virus (DENV), even in countries with temperate climates. The presentation offered a detailed review of the Dengue virus, from its structure to its epidemic potential in Europe and Italy, including molecular, diagnostic, epidemiological, environmental, and preventive features. DENV, which has four known serotypes (DENV-1, DENV-2, DENV-3, and DENV-4), is the arbovirus (of the *Flaviviridae* family) that causes dengue disease. The infection is transmitted to humans by mosquitoes of the genus *Aedes*, whose vector species is *Aedes aegypti*, although transmission is now attributable to the species *Aedes albopictus*, as well as *japonicus* and *koreicus*, invasive species native to East Asia, which have spread to North America, Europe, and now also Italy. Dengue fever is mainly endemic in Africa, Asia, and Central and South America, and DENV, like the Zika and Chikungunya viruses, is considered an emerging virus. Depending on the infecting serotype and its severity, the disease is usually divided into two clinical manifestations: a more severe form in the case of secondary infections with DENV-1 serotype in a subject previously infected with DENV-2 or DENV-3, while uncomplicated dengue generally shows mild symptoms. In her presentation, Prof. Barzon outlined the biology of DENV, a positive RNA virus with an icosahedral capsid that acquires its envelope through budding from the endoplasmic reticulum (ER). The surface of the virion contains the envelope glycoprotein E, whose mature conformation is responsible for the recognition and binding of the virus to specific receptors on the surface of host cells (immune cells and endothelial cells), triggering infection. The difference between mature and immature viral particles is crucial, as only mature virions expose E protein epitopes that are crucial for the development of vaccines targeting the synthesis of neutralizing antibodies. Currently, there is no specific treatment for DENV, which is generally supportive based on the severity of clinical manifestations, but since 2022, a live/attenuated tetravalent vaccine that protects against all DENV serotypes (TAK-003 or DENVax, sold under the brand name Qdenga) has been available in Europe and parts of the world, excluding the US [7]. This vaccine is 61% effective in preventing infection and 84% effective in preventing hospitalizations, with an expected coverage of 7–10 years. In non-endemic settings, a multimodal diagnostic approach is recommended to detect the non-structural protein NS1 and viral RNA by RT-PCR during the acute phase (~7–10 days). Thanks to the presence of IgM and IgG, serological testing guides the diagnosis, which is confirmed by PRNT, especially in the presence of suspected cross-reactivity with other flaviviruses such as West Nile virus (WNV), since in co-circulating contexts or in populations with previous infections or vaccines directed against other flaviviruses, standard serological methods can generate false positives [8]. A central section was devoted to climate change and the geographic expansion of *Aedes albopictus*, encouraged by global tourism, which has led to an increased risk of dengue in temperate countries. In 2024, 693 cases were confirmed in Italy, of which 213 were autochthonous, two and a half times more than in the previous year. The systematic review conducted by Cattaneo et al. 2025 also showed a 25% annual increase in local arbovirus transmission [9]. Prof. Barzon then listed the autochthonous outbreaks that occurred in Italy: in 2020 in Vicenza (DENV-1), in 2023 in Rome and Lombardy (DENV-1 and DENV-3) [10,11], and the 2024 outbreak in Fano (DENV-2) with 199 cases [12], demonstrating a progressive tropicalization of the territory that favors the sustained transmission of DENV, historically typical of tropical areas [13]. In fact, epidemiological-entomological analyses have shown that in Italy, the potential for transmission of the infection is R_0_ ≈ 1.3 and that approximately 90% of secondary transmissions occurred within 500 m of the source of the outbreak, thus supporting the idea that targeted interventions at the local level can limit and potentially block the spread of the virus by directly attacking the vector. Finally, Prof. Barzon highlighted the recent strengthening of European laboratory infrastructure with the establishment of the EURL PH VBV (EU Reference Laboratory for Public Health on Vector-borne Viral Pathogens), a European reference laboratory that has been operational since 1 January 2025, and is coordinated by the RIVM in the Netherlands. The goal of this laboratory is to provide accurate and rapid diagnosis across Europe, standardize laboratory techniques, and improve the overall capacity to respond to arbovirus threats. Finally, Prof. Barzon’s lecture demonstrated that Dengue is no longer a threat limited to tropical areas and that the succession of confirmed epidemics in Italy requires a multisectoral response that integrates molecular, entomological, and vaccine approaches to enable virologists to respond rapidly and effectively to future dengue challenges in Europe.

In the same Session 2, Prof. Maria Rosaria Capobianchi (Department of Infectious, Tropical Diseases and Microbiology, IRCCS Sacro Cuore Don Calabria Hospital, Italy) presented a Lecture titled “Ebola as a paradigm of deadly virus pathogenesis”. The Ebola virus (EBOV) is a paradigmatic model for the study of lethal viral pathogenesis because of its high mortality rate, which can reach 90%, and systemic involvement of vital organs like the spleen, liver, kidney, and vascular system. Near the Ebola River, which gives the disease its name, in the Democratic Republic of the Congo, Ebola viral disease (EVD) was initially identified in 1976. Interhuman spread happens when infected people’s blood, secretions, organs, or other bodily fluids come into direct touch with humans, while transmission to humans happens when humans come into contact with wild animals including fruit bats, porcupines, and nonhuman primates, while interhuman spread occurs through direct contact with blood, secretions, organs, or other bodily fluids of infected individuals, or through surfaces contaminated with such fluids [14]. There is not any proof of aerogenic transfer between people as of yet. According to pathogenetic theory, the infection initially affects dendritic cells and monocytes/macrophages before moving on to the liver, spleen, lungs, and central nervous system. The cytokine storm causes “bystander” death in lymphocytes, but necrosis and vacuolization in infected cells compromise the immune response. According to Schieffelin et al. (2014), hemorrhagic signs are very uncommon, but common clinical symptoms include fever above 38 °C, vomiting, and diarrhea, all of which are frequently linked to an unfavorable outcome [15]. In individuals who have passed away, metabolic acidosis, electrolyte abnormalities, and septic shock are commonly noted. The significance of early and focused treatment based on rehydration, metabolic monitoring, and prompt injection of antivirals and monoclonal antibodies was underlined by Prof. Capobianchi. In a study conducted by Colavita and colleagues (2019) on 44 patients, it was observed that in deceased subjects, viremia was associated with a pronounced inflammatory response, with increased CSF1, IL-6, CXCL1 and CCL2, while in survivors there was an early antibody response, with IgM and IgG production within the first 12 days, associated with control of viral replication [16]. These data underline the critical role of the balance between humoral response and containment of the cytokine storm. An important finding that emerged during the 2014–2016 outbreak in West Africa concerns the persistence of viral RNA in body fluids. In some cases, the virus was isolated from infected cells up to 82 days after clinical onset [17], while viral genetic material was detected up to more than 500 days after the end of infection. Such persistence has facilitated documented episodes of symptomatic reactivation and secondary transmission, confirming that EBOV can persist in immunoprivileged sites, such as the eye, testes, and central nervous system, with the risk of reactivation of infection or delayed sexual transmission. A key aspect of the presentation was the virus’s ability to remain active in survivors; indeed, in patients with post-Ebola uveitis, the virus could be isolated from the ocular fluid (aqueous humor), while in subjects with post-acute meningitis, EBOV was detected in the cerebrospinal fluid [18]. These data suggest that the disease does not necessarily occur following new exposure but can emerge from endogenous viral reservoirs. Next, Prof. Capobianchi described in detail the main pathogenetic mechanisms of the virus: direct damage caused by viral replication, responsible for systemic dissemination and activation of the inflammatory response (with production of TNF-α, IL-1, IL-6, and MIP-1α/β); vascular damage, due to loss of endothelial integrity, coagulopathy, and development of disseminated intravascular coagulation; and finally, the ability of the virus to evade the immune system. Clinical data obtained on two Italian EVD patients treated at INMI Spallanzani [19] showed a novel immunopathogenic mechanism induced by EBOV. Early CD4^+^ cell depletion, accompanied by massive T-cell activation, with increased expression of the depletion marker PD-1 and reduced IFN-γ production, was observed in infected subjects. Gradual recovery of immune function occurred only in the convalescent phase. Finally, Prof. Capobianchi highlighted three key aspects that emerged from the study of EBOV infection: (i) the active nature of viral persistence, as opposed to the classical concept of latency; (ii) the need for long-term monitoring of survivors, especially in immunoprivileged compartments and well beyond the acute phase; and (iii) the related public health implications, as survivors can serve as reservoirs for the virus’s reemergence and trigger new epidemics, making clinical, virological, and sexual follow-up essential. In conclusion, the Lecture offered an integrated and up-to-date view of EBOV as a model of high-lethality viral infection. Combining molecular biology, clinical data, and recent epidemiological observations, Prof. Capobianchi demonstrated how the knowledge gained from the study of Ebola is critical to address future emerging viral epidemics.

**Figure 2:**
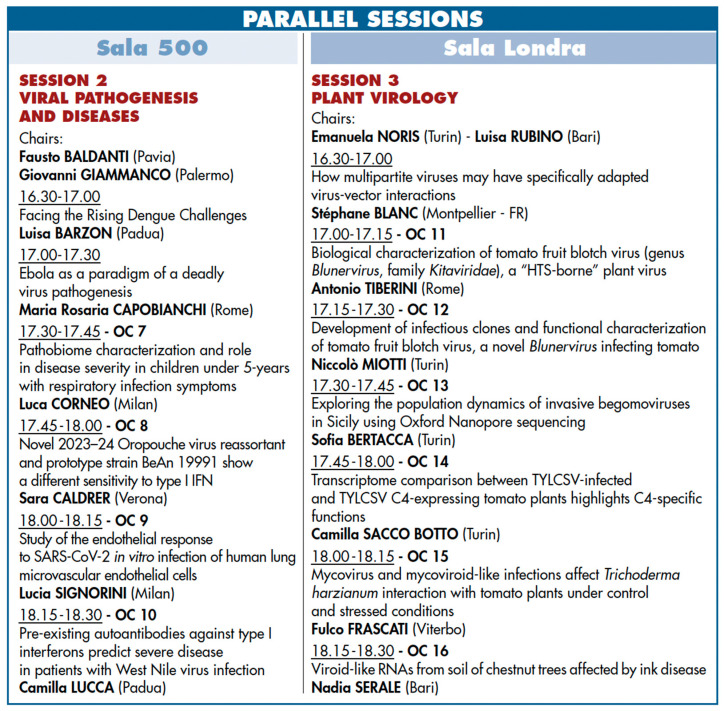
Sessions 2 and 3.

### 2.3. Session 3: Plant Virology—Chaired by Emanuela Noris and Luisa Rubino (Figure 2)

The “Plant Virology” session opened with a talk by Prof. Stéphane Blanc (PHIM, Université Montpellier, IRD, CIRAD, INRAE, Institut Agro, France) on multipartite viruses, which represent one of the most intriguing and conceptually challenging architectures in virology. In these systems, the viral genome is divided into multiple nucleic acid segments, each encased in a separate particle, so that the complete genetic information can only be reconstituted at the population level. Classical theoretical models predict that as the number of genome segments increases, the probability of all of them being transmitted together to a new host becomes extremely small, rendering such viruses nonviable [20]. Considering this evidence, multipartite viruses represent nearly a fifth of all known viral species, suggesting the existence of evolutionary strategies that compensate for the inherent risks of genome fragmentation [21,22].

In this context, Stéphane Blanc and his team have undertaken an elegant experimental exploration of multipartite activity using a nanovirus with eight genomic segments. Their results reveal that the relative abundance of viral segments varies depending on the host plant, suggesting a mutation-free transcriptional regulation mechanism through gene dosage. The results highlight an unexpected level of flexibility in the control of gene expression among multipartite viruses [23]. Even more strikingly, the study demonstrates that spatial colocalization of all genome segments within a single cell is not a mandatory requirement for viral functionality. Instead, distinct genes can act cooperatively at the supracellular level, allowing the virus to maintain systemic infectivity even when individual cells contain incomplete genome sets [23].

Equally innovative is the observation that natural transmitters of nanoviruses (aphid vectors) do not need to acquire all genome segments simultaneously. Different segments can be acquired separately from distinct infected plants, either by a single vector in successive feeding events or by multiple vectors and subsequently reassembled into a complete and functional genome in a new host. This finding suggests a remarkable adaptive capacity in virus-vector interactions, potentially involving mechanisms that increase the likelihood of genome reconstitution despite spatial and temporal separation of viral segments.

### 2.4. Session 4: Virus–Host Interactions—Chaired by Arianna Calistri and Giorgio Gribaudo (Figure 3)

The session “Virus-Host Interactions” started with Prof. Wolfram Brune (Leibniz Institute of Virology, Hamburg, Germany), who held a lecture on “Apoptosis, necroptosis, and pyroptosis: many ways for a cell to die and for a virus to interfere” in which he reminded the audience of apoptosis, necroptosis, and pyroptosis as three important programmed cell death modalities (PCD). Particularly, he focused on the different signaling pathways involved in inflammation and in programmed cell death [24], with attention on the well-known and studied Cytomegaloviruses (CMVs). Regarding this, Prof. Brune summarized how human and murine CMVs interfere with cell death in many ways [25]. Particularly, he described that viral inhibitor of caspase-8-induced apoptosis (vICA) M36 and UL36 in MCMV and HCMV, respectively, inhibit extrinsic apoptosis by blocking Caspase-8 activation; while viral mitochondrial inhibitor of apoptosis (vMIA) and viral inhibitor of Bak oligomerization (vIBO) inhibit intrinsic apoptosis by blocking BAX and BAK activation. MCMV inhibits necroptosis by blocking RHIM-dependent RIPK3 activation, while HCMV inhibits necroptosis by blocking MLKL activation. Finally, MCMV inhibits pyroptosis by interacting with the inflammasome.

The second speaker, Prof. Mirko Cortese (Department of Environmental, Biological and Pharmaceutical Sciences and Technologies, University of Campania Luigi Vanvitelli, Italy) held a lecture on “Molecular architects: how +RNA viruses restructure the cell”, in which he explained the capability that positive-sense single-stranded RNA (+RNA) viruses possess to remodel the host endomembrane system during infection. The virus must have an environmental system for viral replication. Particularly, he described the most striking among the alterations induced by the infection of +RNA viruses, represented by the formation of the viral replication organelle (RO), a specialized membrane-delimited organelle where the viral genome replication takes place [26]. In this regarding he showed that the remodeling of the host cells might contribute to the cytopathogenic effect observed during SARS-CoV-2 infection. In fact, there are two morphotypes of viral replication organelles: an invaginated spherule and a double-membrane vesicle. Using a CryoEM, he analyzed the density of the viral proteins that act as a pore or a platform for the recruitment of other structural proteins in the vesicle lumen to study the remodeling, also in the Zika virus and other flaviviruses.

**Figure 3:**
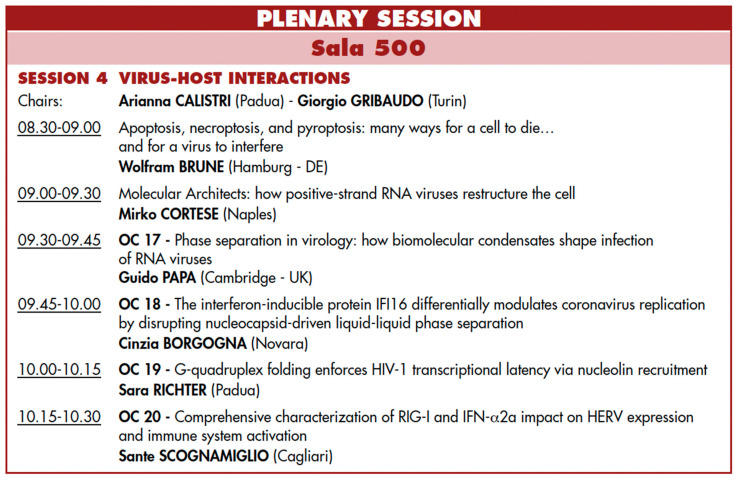
Session 4.

### 2.5. Session 5: Structural Virology and Biotechnology—Chaired by Massimiliano Galdiero and Anna Luganini (Substituting for Giuseppe Portella) (Figure 4)

The session “Structural virology and biotechnology” started with the lecture “Virus vectored genetic vaccines: what have we learnt from pre-clinical and clinical experience” delivered by Prof. Alfredo Nicosia (Department of Molecular Medicine and Medical Biotechnologies, University of Naples Federico II, Italy). Prof. Nicosia opened by framing viral vectors as now-mature platforms for genetic vaccination, whose development accelerated markedly during the COVID-19 pandemic. He reviewed the biological and engineering features that determine vector performance, comparing adenoviral and poxviral (notably MVA) systems [27,28,29]. For adenoviral vectors, he highlighted strong single-dose immunogenicity, robust induction of both neutralizing antibodies and CD8^+^ T cells, and discussed how preexisting anti-adenovirus immunity and vector persistence complicate booster strategies and have driven the adoption of heterologous prime-boost regimens [30]. Turning to poxvirus-derived vectors, Prof. Nicosia emphasized the exceptional safety profile of replication-deficient platforms such as MVA and their capacity to elicit potent cellular immunity, making them well-suited for prime-boost combinations and cancer vaccine applications. He reviewed preclinical data demonstrating how vector backbone choice, capsid or envelope modifications, promoter selection, and antigen design (full-length versus engineered immunogens) modulate antigen expression kinetics, antigen presentation, and the balance of humoral versus cellular responses.

Prof. Nicosia described rational vector engineering approaches his group has pursued: attenuation strategies to minimize reactogenicity, capsid and transgene cassette optimization to enhance dendritic-cell targeting and cross-presentation, and use of molecular adjuvants encoded within vectors to shape innate signals. He illustrated how route of administration and formulation influence tissue tropism and mucosal versus systemic immunity, citing preclinical models where intranasal or intramuscular delivery produced distinct immunoprofiles [31,32]. Prof. Nicosia reviewed clinical experience, stressing that trials of adenoviral-vectored COVID-19 vaccines validated many preclinical predictions on efficacy, safety, and logistic advantages while also revealing rare but important adverse events that necessitate refined screening and risk mitigation. He discussed MVA-based candidates’ favorable tolerability and pronounced T-cell readouts in clinical studies, noting their particular promise for therapeutic cancer vaccination [33,34]. Importantly, he summarized evidence from oncology trials where viral vectors, used alone or combined with immune checkpoint inhibitors, induced tumor-specific T-cell responses and, in several cases, early signals of clinical activity [35]. He also discussed strategies to circumvent anti-vector immunity, including the use of rare human or non-human serotypes, capsid chimerization, and heterologous prime-boost regimens, all supported by both preclinical and clinical data.

In conclusion, Prof. Nicosia presented a balanced synthesis: decades of preclinical and clinical work, culminating in the pandemic-era deployments, confirm adenoviral and poxvirus vectors as mature, adaptable vaccine platforms. Continued engineering, improved manufacturing, and better integration with immunomonitoring and combination therapies, he argued, will further expand their role across infectious disease prevention and cancer immunotherapy.

The presentation by Prof. Ignacio Fernández (Institut Pasteur, Université Paris Cité, Unité de Virologie Structurale, France) reported high-resolution structural and functional studies that illuminate how foamy virus (FV) envelope glycoproteins mediate receptor engagement and membrane fusion, with implications for viral evolution and vector design. Prof. Fernández began by contextualizing FVs as ancient spumaretroviruses with distinctive molecular biology relative to orthoretroviruses (e.g., HIV) and noted their endemicity in non-human primates and zoonotic potential, features that may encourage their development as gene-therapy vectors [36,37]. Despite extensive knowledge of HIV Env, the FV Env architecture and fusion mechanism remained poorly characterized. The group led by Fernandez solved the first crystal structure of an FV receptor-binding domain (RBD), revealing a novel two-subdomain fold; structure-guided mutagenesis identified specific RBD residues required for binding to heparan-sulfate and for infectivity in cell-based assays, establishing the molecular determinants of initial attachment [38]. Complementary cryo-EM single-particle analyses yielded high-resolution reconstructions of the Env ectodomain in both pre-fusion and post-fusion conformations; these structures defined inter-subunit contacts and intramolecular interactions that stabilize Env in a metastable pre-fusion state and traced the large-scale rearrangements that drive membrane merging [36]. Strikingly, comparative structural analyses disclosed unexpected homology between FV Env and fusion glycoproteins from phylogenetically distant virus families, including pneumoviruses, paramyxoviruses, and coronaviruses—suggesting convergent or ancient evolutionary relationships among viral fusogens and highlighting shared mechanistic principles of membrane fusion [39]. This observation supports maintaining FVs in a distinct retroviral subfamily while revealing broader structural parallels relevant to classification and mechanistic inference.

Prof. Fernández emphasized how the structural snapshots clarify potential targets to block entry and reduce zoonotic risk [38]. Methodologically, the combination of X-ray crystallography for the RBD and cryo-EM SPA for full ectodomain assemblies illustrated a powerful pipeline for dissecting envelope glycoprotein dynamics [36,38]. In conclusion, the talk described pre- and post-fusion architectures that explain fusion activation and reveal broader evolutionary connections. These findings advance basic understanding of an ancient retroviral fusogen and provide actionable insights for therapeutic vector design, entry inhibitors, and future structure-function studies.

**Figure 4:**
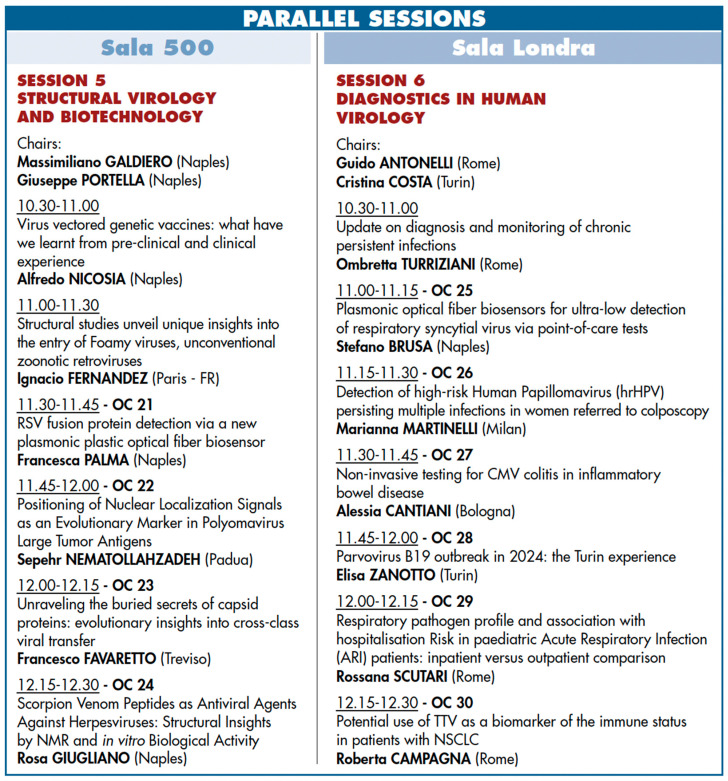
Sessions 5 and 6.

### 2.6. Session 6: Diagnostics in Human Virology—Chaired by Guido Antonelli and Cristina Costa (Figure 4)

Prof. Ombretta Turriziani (Department of Molecular Medicine, University of Rome “La Sapienza”, Italy) held a lecture on “Update on diagnosis and monitoring of chronic persistent infections”.

In her keynote lecture, Prof. Turriziani gave an update on the evolution of diagnostic and monitoring strategies for persistent chronic viral infections, such as those established by HIV, HBV, and HCV. These viral infections are characterized by viral reservoirs, consisting of infected cells or viral genetic material that persists despite antiviral therapy, thus influencing long-term clinical outcomes for patients.

In recent decades, diagnostic virology laboratories underwent profound transformations driven by the introduction of advanced molecular technologies, the development of novel antiviral agents, and the growing need for detailed information concerning viral load, virus genotypes, and antiviral resistance.

The lecture traced the shift from canonical serological testing—such as ELISA and Western Blot—to more sophisticated molecular methodologies. In the past, methods including PCR, quantitative PCR, and nucleic acid testing have allowed for earlier and more sensitive detection of HIV RNA. However, recent innovative techniques—such as CRISPR-based diagnostics, biosensor platforms, and lab-on-a-chip systems—further enhance rapidity.

The quantification of plasma HIV-RNA is essential for clinical monitoring of patients; nevertheless, HIV-DNA represents a marker of viral reservoir. Digital droplet PCR has significantly improved the ability to detect residual viremia, although its routine clinical use is constrained by cost, technical complexity, and limited throughput. While the next-generation sequencing approach provides precious insights into minority drug-resistant quasi-species, this approach still has to overcome barriers related to cost, infrastructure, and the need for standardized data interpretation.

For HBV infection, standard markers including HBsAg, anti-HBc, HBeAg, anti-HBe, and HBV-DNA are of high importance for diagnosis and follow-up of patients. However, these markers are not always able to fully predict the disease progression or therapeutic outcomes. Recently, new biomarkers have proved to be useful for a more exhaustive picture of viral activity. For instance, HBsAg isoforms may represent a promising predictor of the probability to achieve successful cure, even if current analytical limitations preclude routine implementation; HBV core-related antigen may describe the activity of intrahepatic covalently closed circular DNA (cccDNA), also when HBV-DNA in plasma is undetectable, thus HBcrAg is emerging as a marker for monitoring treatment response, predicting relapse, and assessing the risk of liver disease progression; serum HBV-RNA, reflecting active transcription from cccDNA, may represent a promising non-invasive marker of the viral reservoir. Integrated HBV-DNA represents the main contributor to HBsAg production and is implicated in hepatocarcinogenesis. However, its detection requires complex techniques, thus being inapt for routine diagnostics.

The lecture highlighted the need for the introduction of emerging technologies into the healthcare system, along with closer cross-disciplinary collaboration between clinicians and diagnostic virologists.

In conclusion, the diagnosis and monitoring of persistent chronic viral infections are rapidly evolving. The advances achieved represent an important step forward in chronic viral management. These changes are crucial for infection control, thus enabling earlier identification of patients at higher risk of complications, prediction of treatment response, and accurate monitoring of viral reservoirs, thus paving the way for new therapeutic strategies aimed at reaching a successful cure or viral suppression.

### 2.7. Session 7: SIV-ISV/AMCLI Joint Session: The New Frontiers of Virus Sequencing—Chaired by Rossana Cavallo and Tiziana Lazzarotto (Figure 5)

The Joint Session entitled “The New Frontiers of Virus Sequencing” opened with the plenary lecture “Genomics on Human Respiratory Viruses: SARS-CoV-2, Influenza, RSV” delivered by Prof. Paola Stefanelli (Department of Infectious Diseases, Istituto Superiore di Sanità, Italy). Prof. Stefanelli provided an overview of the major applications of genomic sequencing in respiratory virus research, emphasizing its pivotal role in identifying genetic variations and host factors that influence viral infectivity and pathogenesis. She highlighted how genomic surveillance enables real-time monitoring of viral evolution, the emergence and spread of new lineages, and the assessment of public health interventions. Particular attention was devoted to the importance of early genomic warning signals for anticipating outbreaks and designing rapid countermeasures. Using SARS-CoV-2 as a paradigmatic example, Prof. Stefanelli illustrated how the analysis of mutational patterns and the tracking of variants associated with increased transmissibility or immune escape have allowed scientists to integrate genomic and epidemiological data for effective surveillance. This approach is especially crucial for viruses with high mutation rates, such as influenza. Due to frequent recombination and reassortment events, influenza viruses undergo antigenic drift and shift, requiring dedicated surveillance systems. In Italy, the Respivirnet Flu Network, coordinated by ISS, involves 27 laboratories across the country and is supported by the I-Flu-Gen genomic platform for infectious disease surveillance. This infrastructure continuously monitors the circulation of influenza and other respiratory viruses with pandemic potential, characterizing their genetic and antigenic diversity to inform annual vaccine updates. Prof. Stefanelli also discussed the need for an effective genomic surveillance for the respiratory syncytial virus (RSV), a leading cause of hospitalization in infants under one year of age. Given that different RSV genotypes are associated with distinct clinical profiles, whole-genome sequencing—beyond the diagnostic assessment of the F and G genes—is essential to track viral evolution and guide timely preventive measures. In conclusion, Prof. Stefanelli underscored that robust technical methodologies, adequate personnel training, and adherence to best practices for data sharing and transparency are fundamental to ensuring accurate detection and interpretation of viral mutations. These elements are indispensable for maintaining an effective and trustworthy genomic surveillance system capable of responding rapidly to emerging and potentially harmful viral lineages.

**Figure 5:**
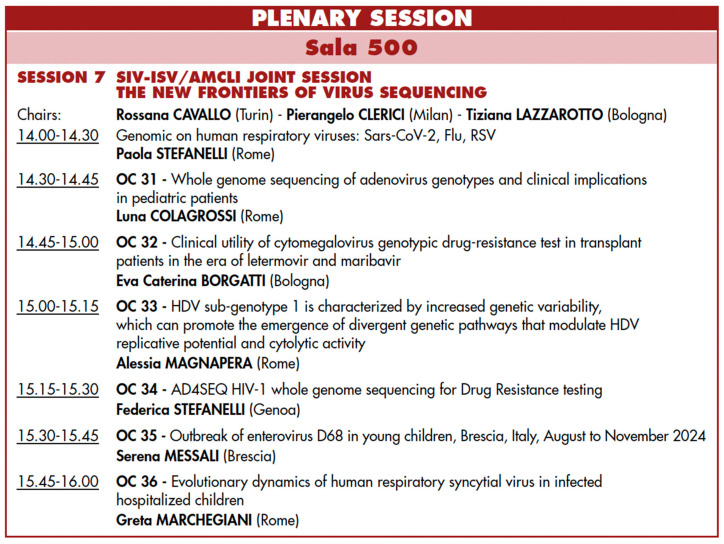
Session 7.

### 2.8. Session 8: Viruses and Cancer—Chaired by: Marisa Gariglio and Aldo Venuti (Figure 6)

The session “Virus and Cancer” was attended by Prof. Nicole Fisher (Institute of Molecular Virology and Tumor Virology, Hamburg, Germany), who discussed “Merkel cell polyomavirus infection and persistence modelled in skin organoids”. She studied this virus, which represents one of the few known human tumor viruses. Due to its direct role in the development of this skin cancer, it is an excellent model for viral tumorigenesis and tumorigenesis in general. Specifically, using a system of skin organoids (SkO) derived from induced pluripotent stem cells (iPSCs) and covered with hair, he demonstrated that they could support viral infection to prove the effectiveness of infection, progression, and long-term persistence [40,41]. Finally, this infection model has enabled us to understand the interactions between viruses and the immune system during infection, test treatment strategies to control reactivation, and map the processes involved in tumor development.

The last guest speaker, Prof. Tim Waterboer (Infections and Cancer Epidemiology, German Cancer Research Center, Heidelberg, Germany) gave a lecture entitled “HPV16 serology-based screening and early diagnosis of HPV-induced oropharyngeal cancer.” After describing the global incidence rate trend, he explained how serological detection of antibodies against HPV16 early proteins, particularly E6, represents a promising strategy for identifying people at risk of HPV-related oropharyngeal cancer (HPV-OPC) [42]. In this context, he highlighted the biological and clinical relevance of HPV16 E6 antibodies, which can appear years before the development of cancer. He reported that solid prospective evidence has been provided that HPV16-E6 seropositivity, although rare in the general population (<1%), is highly predictive of subsequent HPV-OPC, with a risk of up to 25% in 10 years in men [43] in addition to the fact that HPV16 can successfully detect early and asymptomatic cases of HPV-OPC [44], demonstrating that population-based screening using HPV16 serology can successfully detect early and asymptomatic cases of HPV-OPC. Finally, it showed that HPV16-E6 antibodies have high specificity and long-term stability, making them ideal for risk stratification and targeted surveillance, offering a powerful opportunity for secondary prevention and early detection of HPV-driven oropharyngeal cancer, potentially shifting diagnosis toward earlier, more curable stages.

**Figure 6:**
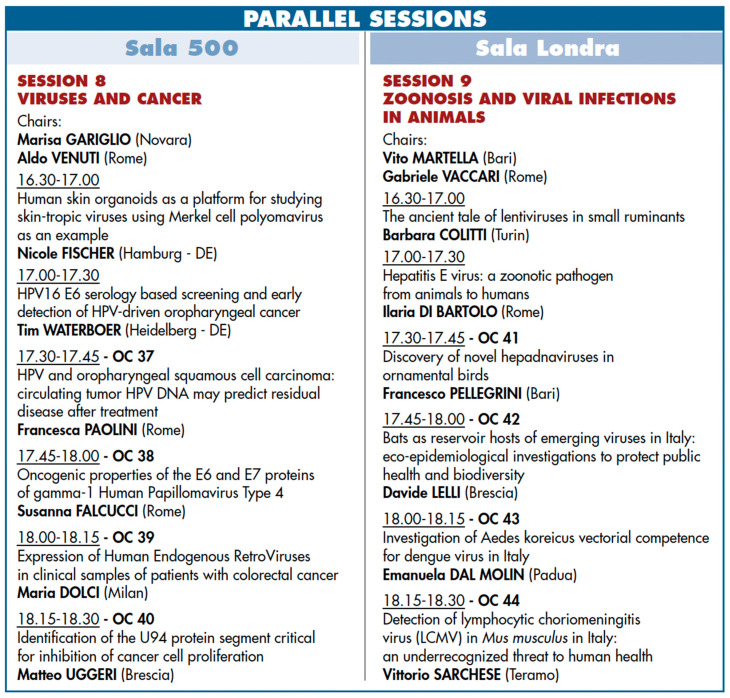
Sessions 8 and 9.

### 2.9. Session 9: Zoonosis and Viral Infections in Animals—Chaired by Vito Martella and Gabriele Vaccari (Figure 6)

Prof. Barbara Colitti (Department of Veterinary Sciences, University of Turin, Italy) reviewed the biology, epidemiology, and evolutionary history of small ruminant lentiviruses (SRLVs), highlighting their global distribution and long-term impact on goat and sheep health and productivity. Emphasis was placed on the multisystemic disease spectrum, pneumonia, encephalitis, mastitis, arthritis, and wasting that SRLVs induce and on the socioeconomic burden for intensive dairy systems, where close contact favors horizontal and vertical transmission. Prof. Colitti traced the viruses’ dissemination through both modern trade and ancient domestication routes, arguing that the current phylogeographic structure reflects millennia of host movement from the Fertile Crescent into Europe and North Africa. Molecular phylogenetics reveals extensive genetic and antigenic heterogeneity driven by high reverse transcriptase mutation rates, recombination, and host-movement patterns [45]. Mediterranean populations show particularly complex histories characterized by multiple introductions and recombination hotspots. This genetic plasticity undermines diagnostic performance; in fact, Prof. Colitti highlighted cases where divergent or recombinant strains escape standard serological and PCR assays due to mutations in immunodominant epitopes, producing false negatives and complicating control. Drawing on her own work, Prof. Colitti outlined advances in molecular surveillance, including sequence-based typing and quasi-species analyses that improve detection of divergent lineages and track recombination events.

Prof. Colitti concluded by underlining research priorities: expanded genomic surveillance with open, shared sequence databases; development of broadly reactive diagnostics and standardized classification schemes; studies on host genetic resistance and immune correlates. Prof. Colitti highlighted that only by integrating evolutionary, molecular, and field epidemiology can durable, context-appropriate SRLV control be achieved [46,47].

The second invited speaker of the session, Dr. Ilaria Di Bartolo (Department of Food Safety, Nutrition and Veterinary Public Health, Unit of Emerging Zoonoses, Istituto Superiore di Sanità, Italy), reviewed hepatitis E virus (HEV) biology, epidemiology, and food-safety implications with emphasis on genotype 3 (HEV-3) as a zoonotic threat in Europe. Dr. Di Bartolo summarized surveillance and source-attribution data showing domestic pigs and wild boar as principal reservoirs, and documented outbreaks and sporadic human cases linked to consumption of raw or undercooked pork liver products and wild game [48]. She reported the widespread detection of HEV RNA and high seroprevalence across the swine production chain—on farms, at slaughterhouses, and in retail pork products (liver, sausages, pâtés), highlights the pervasive contamination risks in the food supply. Dr. Di Bartolo highlighted methodological advances enabling recent progress: validated molecular assays for detection, sequencing for phylogeographic tracing, and, crucially, the advent of robust cell-culture systems that permit viral infectivity assessment [49]. Using these culture systems, evidence has shown that HEV-3 can survive common food-processing conditions, displaying partial resistance to heating, tolerance to prolonged freezing, and persistence at ambient storage [50], thereby refining risk assessments for culinary practices involving raw or minimally processed pork products. She further emphasized genotype-dependent disease severity, noting that immunocompromised patients and those with underlying liver disease are at particular risk of chronic infection and severe outcomes.

Dr. Di Bartolo concluded that HEV-3 represents a paradigmatic One Health pathogen: its control requires molecular epidemiology, infectivity assays, coordinated surveillance, and pragmatic interventions throughout the food chain [51]. Continuous monitoring, improved diagnostics, and cross-sector collaboration, she argued, are essential to reduce zoonotic transmission and prevent human cases linked to contaminated pork and wild game products.

### 2.10. Session 10: Antiviral Therapy—Chaired by David Lembo, Mauro Pistello, and Enzo Tramontano (Figure 7)

The plenary Session 10 devoted to “Antiviral Therapy” was opened by the Lecture of Prof. Pierre-Olivier Vidalain (CIRI, Centre International de Recherche en Infectiologie, Université Claude Bernard Lyon 1, France). Prof. Vidalain clearly illustrated how fundamental cellular metabolism is in the context of viral infections and the potential therapeutic strategies involved. Over the past decade, findings from metabolomics studies have revealed how viruses are able to significantly alter host cell metabolism. Indeed, they activate metabolic pathways such as glycolysis, lipogenesis, and NAD(H) synthesis to ensure the energy and precursors needed for effective viral replication [52]. To overcome the drawbacks of the present direct antiviral medicines (DAAs), Prof. Vidalain noted that focusing on cellular metabolism may be a viable approach for the creation of broad-spectrum antivirals, concentrating especially on: (i) metabolic enzymes, (ii) metabolic pathways, and (iii) metabolites that are thought to be antiviral agents. Acute infection-causing viruses like Dengue, Influenza, and SARS-CoV-2, as well as chronic infection-causing viruses like HBV and HCV, require particular metabolic pathways to aid in their reproduction. Hexokinase 2 (HK2), one of the essential enzymes of glycolysis, is directly bound by the HCV viral protein NS5A, which increases its activity [53,54]. This binding promotes the development of viral lipo-viroparticles by facilitating the uptake of glucose, the synthesis of lactate, and the process of lipogenesis. Concurrently, the PI3K/Akt and JNK pathways also promote gluconeogenesis, and the transcription factors FoxO1 and HNF-4α facilitate the upregulation of PEPCK and G6Pase. Acute viruses, including Dengue, Influenza, and SARS-CoV-2, exhibit a similar behavior, early activation of both lipid and glycolysis pathways. In the particular instance of Dengue, the regulatory protein GCKR, which typically suppresses glucokinase (GCK), interacts with the viral protein NS3 to increase hepatic glycolysis. The activation of GCK by the binding of NS3 to GCKR promotes glycolysis and the NAD(H) pathway, offering a promising pharmaceutical avenue to combat viral infections [55]. Prof. Vidalain pointed out that targeting cellular metabolism could be a promising strategy for the development of broad-spectrum antivirals, overcoming the limitations of current direct antiviral therapies (DAAs). Specifically focusing on: (i) enzymes involved in metabolism, (ii) metabolic pathways, and (iii) metabolites understood as antiviral agents. Viruses responsible for acute infections, such as Dengue, Influenza, and SARS-CoV-2, as well as those causing chronic infections such as HBV and HCV, exploit specific metabolic pathways to facilitate their replication. A significant example is HCV viral protein NS5A, which binds directly to hexokinase 2 (HK2), one of the key enzymes of glycolysis, increasing its activity [54]. This binding facilitates glucose uptake, lactate production, and lipogenesis, events that contribute to viral lipo-viroparticle formation. Simultaneously, gluconeogenesis is also stimulated via the PI3K/Akt and JNK pathways, with increased expression of PEPCK and G6Pase mediated by the transcription factors FoxO1 and HNF-4α. A similar phenomenon is observed in acute viruses: Dengue, Influenza, and SARS-CoV-2, which activate both glycolysis and lipid pathways early. In the specific case of Dengue, hepatic glycolysis is enhanced by an interaction between the viral protein NS3 and the regulatory protein GCKR, which normally inhibits glucokinase (GCK). The binding between NS3 and GCKR activates GCK, thereby promoting glycolysis and the NAD(H) pathway, providing an interesting pharmacological opportunity to act against viral infections [55]. Viruses such as HBV and Vaccinia also manipulate NAD(H) metabolism, altering redox levels and synthesis of nucleic acids and lipids. This has sparked growing interest in cell–host-targeted antivirals (HTAs): instead of directly targeting the virus, these therapies modulate metabolic pathways exploited by the pathogen, resulting in broad-spectrum effects and reducing the risk of resistance. Some examples of HTAs include: 6-Aminonicotinamide (6-AN), which inhibits the pentose-phosphate pathway; FK866, which blocks NAD^+^ synthesis [56]; FXR agonists such as vonafexor and GW4064, which modify hepatic lipid metabolism and hinder HBV replication; and BEZ235, a dual inhibitor of PI3K/mTOR, which has been shown to significantly reduce influenza virus infectious particles but does not interfere with the early stages of infection [57]. Another novel approach is to use the metabolites as antivirals, such as succinate, which has shown strong activity against influenza A virus (strains A/H1N1 and A/H3N2), reducing metabolic changes and inflammatory responses related to viral infection. This metabolite induces post-translational succinylation of the viral nucleoprotein (NP) on residue K87, thereby modifying electrostatic interactions with RNA and hindering trafficking of viral ribonucleoprotein complexes [58]. Screening metabolism-oriented compound libraries has also led to the discovery of novel antiviral molecules, such as molidustat, which acts on prolyl-hydroxylases (PHDs) that in turn degrade the transcriptional factor HIF-α, thereby modulating key metabolic pathways (glycolysis, lipogenesis, nucleotide biosynthesis) controlled by HIF-α for viral replication. Although antiviral HTAs may have a wide range of action with less risk of resistance emergence, a more potent therapeutic approach could rely on the synergistic combination of DAAs, which directly target the virus, and HTAs, which act on the cellular metabolic environment. This duplicity not only reduces the risk of resistance development but also provides effective protection against emerging viral variants or strains, representing a significant advance in antiviral therapy.

**Figure 7:**
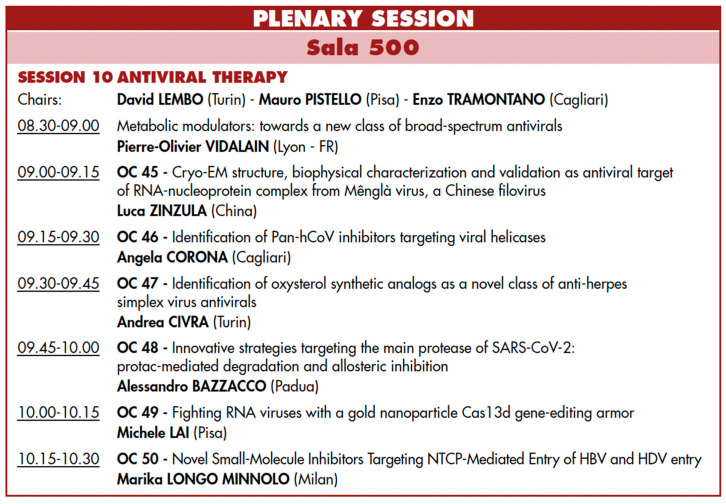
Session 10.

### 2.11. Session 11: Emerging and Re-Emerging Viruses—Chaired by Francesca Caccuri and Clementina Cocuzza (Figure 8)

Prof. Carlo Federico Perno (UniCamillus University—International Medical University, Italy; Microbiology Unit, IRCCS Ospedale Pediatrico “Bambino Gesù”, Italy) held a lecture on “Re-emerging viruses: a journey between pathogens and humans”.

In his keynote lecture, Prof. Perno provided an overview of emerging and re-emerging viral infections, with particular attention to their epidemiology, clinical implications, and diagnostic approaches.

A central subject in his lecture was the multifactorial nature of viral emergence. The COVID-19 pandemic generated alterations in the immunity of the population, which, together with environmental dynamics and pathogen evolution, have contributed to the reshaping of viral circulation patterns. This phenomenon can be exemplified by the atypical off-season peaks of human metapneumovirus (hMPV) observed between 2021 and 2023, soon after the reduction in pandemic mitigation measures [59].

Also, *Bordetella pertussis* underwent a marked resurgence in 2024 after several years of minimal circulation [60]. Prof. Perno discussed how pediatric surveillance data from the Bambino Gesù Children’s Hospital revealed a marked increase in positive tests accompanied by a high incidence of respiratory co-infections, suggesting intricate viral–bacterial interactions within the respiratory ecosystem.

Among the re-surged infections, the theme of parvoviruses has been discussed. In 2023–2024, an uncommon increase in Parvovirus B19 infections has been reported in several European countries [61]. At the Bambino Gesù Children’s Hospital, the incidence of Parvovirus B19 infections increased considerably during 2024. These infections were associated with severe disease manifestations—including myocarditis, encephalitis, and hematologic abnormalities—with some requiring extracorporeal support. The most circulating genotype, as assessed by viral genomic characterization, revealed the exclusive circulation of genotype 1A with low intra-population variability, thus supporting the hypothesis that the epidemiological surge was most likely driven by population dynamics rather than viral genetic diversification [62,63].

Also, infections caused by human adenoviruses (HAdV) were deeply considered due to their clinical relevance in immunocompromised populations. The emergence of recombinant HAdV variants is facilitated by their ability to establish latency within mucosa-associated lymphoid tissues, combined with frequent recombination events in capsid and immune-modulatory genes. The recombinant variants are of particular importance because they exhibit altered tissue tropism and virulence. This event is exemplified by the recombinant strain HAdV-B55, which possesses enhanced replication in respiratory epithelial cells and increased pathogenic potential as compared with its parental strains [64]. Since viral genotype distribution differs between immunocompetent and immunocompromised pediatric patients, it is of particular importance to apply genotype-informed risk stratification.

Prof. Perno also stressed the increasing importance of advanced molecular diagnostics, in particular, metagenomic next-generation sequencing (mNGS). Different from targeted PCR-based assays, mNGS allows unbiased detection of all microbial nucleic acids present in a sample, allowing the identification of unpredicted or novel pathogens, the characterization of microbial populations, and the definition of transmission clusters. Literature data showed the powerful mNGS diagnostic support, as attested by the detection of *Aspergillus oryzae* in a child with persistently negative conventional microbiological tests [65,66,67].

Finally, the integration of artificial intelligence into clinical microbiology has been discussed as an evolving and ground-breaking edge. It provides automated colony quantification, pathogen classification, resistance prediction, and high-throughput analysis of genomic datasets, thus enhancing diagnostic accuracy and laboratory efficiency.

In conclusion, the lecture highlighted the urgent need for a drastic change in pathogen surveillance and diagnostics. Moving from the limited investigations of presumed pathogens, it is fundamental to adopt strategies able to detect the unexpected. Metagenomics and artificial intelligence—integrated with clinical expertise—constitute an essential tool for prompt identification, control, and prevention of emerging and re-emerging viral infections.

**Figure 8:**
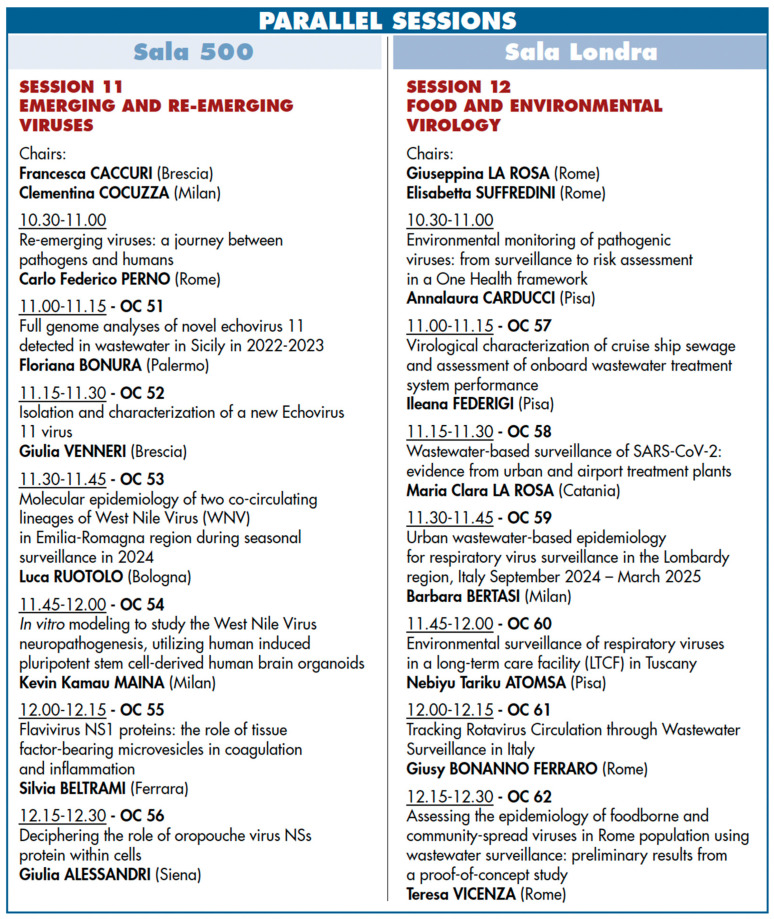
Sessions 11 and 12.

### 2.12. Session 12: Food and Environmental Virology—Chaired by Giuseppina La Rosa and Elisabetta Suffredini (Figure 8)

Session 12 was focused on environmental viral monitoring that emerged as a critical component of One Health strategies, linking human, animal, and ecosystem health. Prof. Annalaura Carducci (Laboratory of Hygiene and Environmental Virology, Department of Biology, University of Pisa, Italy) emphasized the pivotal role of the environment in viral epidemiology, highlighting its function as a reservoir and conduit for pathogen transmission via water, air, soil, surfaces, fomites, and food. Pathogenic viruses excreted through feces, urine, respiratory secretions, and blood can contaminate these matrices, facilitating both zoonotic and anthroponotic spillover events 1 [68].

Historical evidence, including early poliovirus studies, demonstrated the environmental transmission potential of viruses, but recognition of this phenomenon increased significantly in the late 20th century with emerging outbreaks and the implementation of WHO surveillance [69]. Key viral features—high excretion rates, resistance to inactivation, low infectious doses, multiple transmission routes, and genetic variability—make environmental monitoring essential for risk assessment, exposure evaluation, and control measure effectiveness [70]. Wastewater-based epidemiology (WBE) exemplifies the application of environmental surveillance for both waterborne and non-waterborne viruses, enabling molecular epidemiology, variant tracking, and early-warning detection at the community level [71]. Effective monitoring relies on sample concentration, purification, and standardized process controls, coupled with biomolecular methods such as PCR, digital PCR, and next-generation sequencing. Prof. Carducci underscored the need to integrate technological advances with targeted applications, including exposure risk assessment, wastewater treatment evaluation, and surveillance of endemic and emerging pathogens. These strategies are fundamental for prevention, preparedness, and rapid response to current and future epidemic threats, reinforcing the central role of environmental virology in public health.

## 3. Sponsored Symposia

### 3.1. Sponsored Symposium 1—Chaired by Simona Fiorentini and Giovanni Delogu (Figure 9)

During the Sponsored Symposium with the unrestricted grant of ELITech Group entitled “CMV RNA: translating Diagnostics into Clinical practice—Insights from a Clinical Microbiologist and a Paediatric Gastroenterologist”, Prof. Tiziana Lazzarotto (Department of Medical and Surgical Sciences, University of Bologna, Italy) delivered a lecture entitled “CMV RNA: Translating Diagnostics into Clinical Practice—Insights from a Clinical Microbiologist”. Currently, the quantification of cytomegalovirus (CMV) DNA in blood samples (CMV-DNAemia) represents the gold standard for identifying active viral replication, preventing CMV-related disease, and monitoring response to drugs targeting CMV-DNA polymerase. The recent introduction of letermovir (LMV) showed that CMV-DNAemia may not be an accurate marker of active viral replication. Indeed, by blocking the terminase complex, LMV induces the release of free CMV-DNA fragments (abortive infection), which could lead to potential misinterpretation of molecular testing results. Additional methods, such as CMV-viremia (shell vial method) and CMV-DNAemia post-DNase (DNase test), could be used to prove active viral replication. CMV viremia detects CMV infectious particles in cell culture, whereas the DNase test exploits the digestion activity of DNase I added to the sample before extraction to differentiate free naked DNA from the genome encapsidated into virions. Both methods, therefore, identify the presence of infectious virions in blood samples. However, the procedures are laborious and not standardized. In a study published by Piccirilli et al. (2024), it has been demonstrated that detectable low DNAemia levels during LMV administration may reflect abortive rather than productive infection, suggesting that CMV-RNAemia could be useful for detecting active CMV replication in patients receiving antiviral therapy, especially with drugs that do not act on the viral DNA polymerase, such as LMV and maribavir (MBV) [72]. In addition, CMV-RNAemia UL21.5 allows identification of episodes of active viral replication and not blips of self-resolving DNAemia. The CMV-RNAemia assay is automated and more standardized than the CMV-viremia and DNase assay procedures. Peak viral loads are reached simultaneously, in the downstream phase, negative results are reached earlier with CMV-RNAemia than with CMV-DNAemia, demonstrating more rapid viral clearance [72]. In another study published by Giardina et al. (2025) [73], it has been shown that CMV UL21.5 mRNA is detectable in plasma during clinically significant episodes of CMV DNAemia in immunocompromised patients. The mRNA detected in plasma is mostly virion-encapsidated, and its levels correlate with those of CMV DNAemia. UL21.5 mRNA is absent in the plasma of patients showing transient self- resolving DNAemia blips during LTV prophylaxis (i.e., abortive infection), whereas it is detectable in patients with CMV DNAemia because of CMV infection resistant to LMV or MBV (i.e., productive virus replication) [73]. In the second part of the Symposium, Dr. Emanuele Nicastro (Pediatric Hepatology, Gastroenterology and Transplantation, Hospital Papa Giovanni XXIII, Bergamo, Italy) delivered a lecture entitled “Cytomegalovirus-RNA accurately identifies clinically significant infection needing preemptive therapy in liver transplanted children: a proof-of-concept study”. CMV infection poses a significant risk in solid organ transplantation, especially in pediatric liver transplant (LT) recipients, who are often CMV-naïve. While prophylaxis with ganciclovir or valganciclovir helps prevent early CMV infection, it carries risks like neutropenia and delayed reactivation. Preemptive therapy (PET), which initiates antiviral treatment based on viral load thresholds, is safe and effective in controlling CMV infection after LT. Current PET protocols rely on blood CMV-DNA load to identify subjects likely to develop overt CMV disease. CMV-DNA determination by PCR exhibits high sensitivity, reliability, even in severely neutropenic patients, and short turnaround time. However, the viral load detected as CMV-DNAemia could represent a free viral genome released from cells or tissues rather than infectious viral bodies, as suggested by the relatively longer persistence of CMV-DNA in plasma (i.e., cell-free) compared to whole blood (i.e., cell-free and intracellular) specimens of HSCT and kidney transplant recipients [74,75]. Comparative monitoring of a CMV-specific mRNA transcript has shown promising accuracy in differentiating between true CMV infection and abortive CMV-DNAemia in solid organ transplant and adult HSCT treated with letermovir. CMV UL21.5 mRNA is thought to mark active viral replication in immunosuppressed patients [73]. The team directed by Dr. Nicastro assessed its accuracy in identifying clinically significant CMV infection (csCMVi) needing PET in LT children. Plasma quantitative UL21.5 mRNA (CMV-RNA) and whole blood CMV-DNA were obtained weekly after LT, and patients were followed for 6 months. CsCMVi was defined as the achievement of the criterion for PET (CMV-DNA ≥ 100,000 IU/mL or 50,000 in high-risk children). PET consisted of standard ganciclovir or valganciclovir until negative CMV-DNA. One hundred forty-four quantitative CMV-RNA and CMV-DNA determinations were obtained from 12 children. Of 52 CMV-DNA-positive specimens, 17 (32%) were also CMV-RNA-positive, while CMV-RNA was undetectable in CMV-DNA-negative specimens. All children with csCMVi had early detectable CMV-RNA, peaking simultaneously to CMV-DNA (median CMV-DNA: 65,906 cp/mL; median CMV-RNA: 767 cp/mL); conversely, none of those with persistently low DNAemia proved CMV-RNA-positive. In this first pilot study, CMV-RNA had 100% sensitivity and specificity in identifying children needing PET after pediatric LT [75]. The detection of UL21.5 CMV-RNA clearly identifies children with csCMVi after LT, thus allowing prompt antiviral administration without the risk of overtreatment. CMV-RNA might replace CMV-DNA monitoring in the decision-making of anti-CMV PET.

These findings support the integration of CMV-RNA testing into clinical practice as a valuable tool for improving diagnostic accuracy and guiding targeted, patient-tailored management of CMV infections.

**Figure 9:**
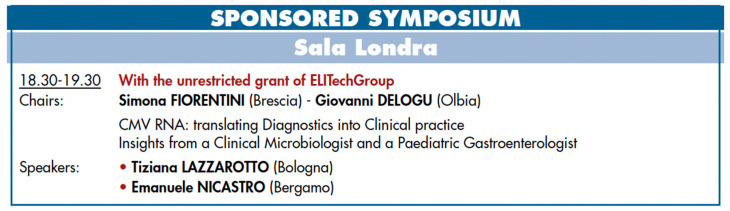
Sponsored symposia 1.

### 3.2. Sponsored Symposium 2—Chaired by Cristina Costa (Figure 10)

The Sponsored Symposium with the unrestricted grant of Diasorin “HDV, a small virus for aggressive hepatitis” opened with a lecture by Prof. Mario Rizzetto (Department of Medical Sciences, University of Turin, Italy) entitled “From Beginnings to Latest Guidelines and Clinical Evidence.” Prof. Rizzetto recalled that HDV is a major hepatotropic pathogen distributed worldwide, characterized by a defective, non-autonomous replication that depends on hepatitis B virus (HBV) co-infection or superinfection. HDV exploits HBV surface antigen (HBsAg), produced both from episomal HBV cccDNA and integrated HBV DNA, to enter hepatocytes through the sodium taurocholate cotransporting peptide (NTCP), the same receptor used by HBV. Once inside the cell, the HDV RNA genome is replicated in the nucleus by cellular RNA polymerase II, and virion assembly occurs through farnesylation-dependent interaction between the large delta antigen (L-HDAg) and HBsAg. From a clinical standpoint, chronic HDV superinfection in asymptomatic HBV carriers leads to clinical worsening in approximately 90% of cases, with up to 70% developing cirrhosis within 10 years. The introduction of HBV vaccination has dramatically changed HDV epidemiology, leading to a marked decline in incidence in high-income countries, while in low-resource regions—particularly parts of Africa and Asia—the burden remains closely linked to HBV endemicity. Diagnosis of HDV infection begins with serological detection of anti-HDV antibodies, followed by confirmatory testing for HDV RNA to assess active viral replication. Historically, testing was limited to patients with advanced liver disease; however, the most recent EASL Clinical Practice Guidelines (European Association for the Study of the Liver, 2025) recommend universal HDV screening in all HBsAg-positive individuals to avoid missed diagnoses [76]. Regarding therapeutic strategies, Prof. Rizzetto emphasized that HDV poses unique challenges due to its replication mechanism: a rolling-circle process driven by host RNA polymerase, making it intrinsically resistant to conventional antivirals targeting viral polymerases (such as for HBV and HCV). Novel targets include the HDV ribozyme and host cellular enzymes such as RNA ligase. Apart from replication players, another antiviral strategy involves the use of entry inhibitors: Bulevirtide (BLV), an HBsAg-derived lipopeptide that binds NTCP with high affinity, blocks HDV entry into hepatocytes. However, its efficacy is limited as it does not inhibit viral replication in already infected cells, where HBsAg continues to be produced from integrated HBV DNA. Moreover, HDV’s high infectivity (even at 10^−11^ serum dilutions) and the persistence of low-level viremia below current detection thresholds (≈6 IU HDV RNA/mL) contribute to post-treatment relapses. Therefore, the only definitive therapeutic endpoint remains HBsAg clearance, a rare event in clinical practice. Surrogate endpoints include sustained virologic response (SVR), defined as long-term undetectable HDV RNA. Recent therapeutic research explores combination regimens and novel agents. The combination of BLV with pegylated interferon (peg-IFN) aims to both block new infections and reduce intrahepatic replication [77]. Other promising strategies directly target HBsAg, such as Tobevibart (VIR-3434), a monoclonal antibody against the conserved antigenic loop of HBsAg, evaluated in combination with Elebsiran (VIR-2218), an siRNA that silences HBV transcripts [78]. Another innovative approach involves BJT-778, a human anti-HBsAg monoclonal immunoglobulin currently under investigation [79].

In the second part of the symposium, Prof. Gian Paolo Caviglia (Department of Medical Sciences, University of Turin, Italy) presented a lecture on “Circulating HDV RNA and Alternative Markers in Patients with Chronic HDV Infection: Ongoing Studies.” He emphasized that chronic HDV infection represents the most severe and progressive form of viral hepatitis, with persistent viremia being the strongest predictor of liver disease progression. In particular, the implementation of reflex screening—i.e., testing all HBsAg-positive individuals for anti-HDV antibodies and HDV RNA—is crucial, having increased diagnostic yield fivefold [80]. Data from the PITER cohort (5494 HBsAg-positive patients from 59 Italian centers, 2019–2023) revealed a 10% national HDV prevalence [81], rising to 84% in Turin in the DESCRIBE Study Cohort (515 patients from 32 centers, 2022–2024) [82]. Prof. Caviglia also discussed diagnostic challenges arising from assay variability and genotype distribution: in Italy, genotype 1 accounts for ~99% of infections, in both natives and immigrants, with genotype 5 detected in ~1% [82]. A national quality-control study compared diagnostic performances of genotype 1 HDV-RNA quantification assays used in clinical practice by 30 Italian centers, including both commercial (*n* = 27) and in-house (*n* = 3) assays. Results revealed assay sensitivity ranging from 3 to 300 U/mL and up to 30% inter-laboratory variability according to the assay used [83]. Additionally, the method of RNA extraction influenced sensitivity, with manual extraction providing ≈1 log IU/mL higher detection [84]. Given these analytical limitations, alternative indicators such as HD antigen (HD-Ag) and anti-HDV antibodies are being explored as potential direct and indirect surrogate markers for HDV serological assessment, respectively. HD-Ag detection faces technical obstacles due to immune complex formation and viral particle shielding, while anti-HDV IgM titers show only moderate correlation with HDV RNA levels. Prof. Caviglia’s group reported preliminary findings in 52 patients demonstrating a strong correlation between total anti-HDV titers and HDV RNA levels (r = 0.718; AUC = 0.908), suggesting promising diagnostic potential. Larger multicenter studies are needed to validate anti-HDV titration as a surrogate marker for active infection and to define clinically meaningful cut-offs for risk stratification, he concludes.

**Figure 10:**
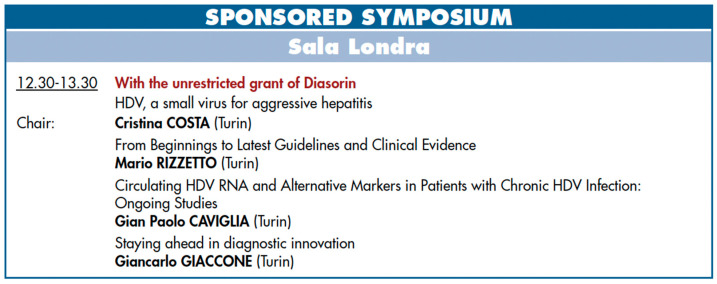
Sponsored Symposium 2.

## 4. Awards and Prizes

SIV-ISV AWARD 2025—Chaired by Arnaldo Caruso

Prof. Paolo Lusso (Laboratory of Immunoregulation and Infectious Diseases, National Institute of Allergy and Infectious Diseases, NIH, Bethesda, USA) delivered a compelling, career-spanning lecture that flowed like a detective story of molecular discovery and translational persistence. He recounted how a young clinician-scientist from Torino with mentors like Robin Foà and Felice Gavosto, he stepped into Robert Gallo’s Laboratory of Tumor Cell Biology (NCI, Bethesda), where his career in human retrovirology took shape and, over three decades, moved the field from fundamental insights about chemokine biology to cutting-edge vaccine design [85]. Prof. Lusso narrated scientific turning points with vivid detail, how a puzzling CD8^+^ T-cell suppressive activity was tracked, characterized and finally resolved into three chemokines (RANTES, MIP-1α, MIP-1β), transforming our understanding of innate antiviral control and revealing the chemokine–coreceptor axis as a central determinant of HIV-1 tropism and pathology [86,87,88]. He illustrated these discoveries with personal recollections of experiments, debates, and collaborations that shaped the era. After a short comeback in Italy in 2006, he moved back to the USA to lead a section at NIAID’s Laboratory of Immunoregulation (at Anthony Fauci’s invitation) broadened his translational focus, bridging mechanistic studies of viral entry and immune regulation with vaccine and therapeutic development.

Prof. Lusso then wove chemokine biology into a broader tapestry of virus–virus and virus–host interactions. He reviewed his group’s work elucidating the interplay between HHV-6 and HIV-1, showing how herpesvirus co-infections modulate HIV replication and immune responses, as well as the identification of cellular receptors for HHV-6 and HHV-7 [89]. He emphasized how these findings sharpened concepts of cellular susceptibility, viral modulation of immune trafficking, and the ecological niches that favor persistence or disease progression.

Turning to receptor biology, Prof. Lusso summarized in vitro and in vivo studies that defined chemokine receptor usage by HIV-1, clarifying the R5/X4 dichotomy and its clinical significance [90]. He described the discovery of a second CD4-binding site on HIV-1 Env, a mechanistic insight that refined models of entry and exposed new vulnerabilities for neutralization and vaccine targeting.

Throughout, he emphasized methodological enablers that shaped his career: identification of molecular correlates of entry and suppression, structural virology of Env, longitudinal cohort studies, neutralization mapping, and systems vaccinology to identify early biomarkers of efficacy. He concluded with a forward-looking perspective that expresses cautious optimism: convergence of improved structural immunogen design, advanced adjuvants, novel delivery platforms, and global trial infrastructures increases the likelihood of achieving progressively effective preventive and therapeutic vaccine strategies against HIV-1 [91,92].

In recognition of his towering scientific record, Prof. Lusso was awarded the SIV-ISV AWARD 2025 for lifetime achievement, a fitting capstone to a lecture that combined rigorous science, human stories, and translational vision.

In line with our commitment to supporting the next generation of virology researchers, three awards were presented to Dr. Sante Scognamiglio (Department of Life and Environmental Sciences, University of Cagliari, Italy), Prof. Luca Zinzula (iHuman Institute, ShanghaiTech University, China), and Dr. Giulia Venneri (Department of Molecular and Translational Medicine, University of Brescia, Italy).

## 5. Concluding Remarks

In summary, the 9th SIV-ISV National Congress successfully combined scientific excellence, translational relevance, and a strong One Health ethos. By convening experts across disciplines and career stages, the meeting fostered the exchange of unpublished and published findings, stimulated debate on pressing methodological and policy questions, particularly around genomics, bioinformatics, and surveillance, and promoted initiatives to support the next generation of virologists. The outcomes of Turin include concrete scientific insights, reinforced networks for collaborative research, and a clarified agenda for future investments in infrastructure, training, and integrated surveillance systems. The Organizing Committee thanks all speakers, chairs, participants, sponsors, and the staff of Centro Congressi Lingotto, and above all, the Nadirex staff for their contributions; the discussions and connections generated at the Congress lay a durable foundation for continued multidisciplinary efforts to understand, prevent, and mitigate viral threats while harnessing viral biology for therapeutic and technological innovation.

## Data Availability

The data that support the findings of this study are contained within the article.
